# Alzheimer’s disease and cerebrovascular biomarkers in relation to odor identification in a naturalistic clinical cohort

**DOI:** 10.1186/s13195-026-02073-w

**Published:** 2026-06-03

**Authors:** Javier Oltra, Zuzana Ištvánfyová, Grégoria Kalpouzos, Ingrid Ekström, Göran Hagman, Miia Kivipelto, Erika J Laukka

**Affiliations:** 1 Aging Research Center, Department of Neurobiology, Care Sciences and Society, Karolinska Institutet and Stockholm University, Stockholm, Sweden; 2 Division of Clinical Geriatrics, Center for Alzheimer Research, Department of Neurobiology, Care Sciences and Society, Karolinska Institutet, Stockholm, Sweden; 3 Theme Inflammation and Aging, Karolinska University Hospital, Stockholm, Sweden; 4 Institute of Public Health and Clinical Nutrition, University of Eastern Finland, Kuopio, Finland; 5 Ageing Epidemiology Research Unit, School of Public Health, Imperial College London, London, UK; 6 Stockholm Gerontology Research Center, Stockholm, Sweden

## Abstract

**Introduction:**

Olfactory deficits, especially in odor identification (OID), have been linked to Alzheimer’s disease (AD), likely due to regional proteinopathy and atrophy in the olfactory brain circuit. Their cognitive and biological correlates across the clinical spectrum, particularly in individuals with no evident cognitive impairment, have been underexplored. This examination is relevant because many individuals of this group will not progress to dementia, and olfactory deficits may reflect ongoing pathological processes and could enrich risk stratification.

**Methods:**

We analyzed data from a cohort of 233 subjective cognitive impairment (SCI, *n*=152), mild cognitive impairment (MCI, *n*=50), and AD dementia (*n*=31) individuals from the Karolinska University Hospital Memory Clinic (Solna, Sweden). We examined the association of performance on the 16-item Sniffin’ Sticks OID test (free and total [free or cued] identification scores) with a range of markers: cognitive performance, cerebrospinal fluid biomarkers, AD- and olfactory-related brain volumes, and white matter hyperintensities volume. We performed correlation analyses, generalized additive models, and threshold regressions, adjusted for sociodemographic factors and *APOE* status.

**Results:**

OID performance was better in SCI compared to MCI and AD. Aβ42/40 ratio was positively associated with OID in SCI and AD, with *APOE* ε4 carriers driving this association in SCI. Hippocampal volume was positively associated with OID in AD. Higher volume of white matter hyperintensities was negatively associated with OID in MCI. The relationships of OID with Aβ42/40 and hippocampal volume were linear in the whole cohort. Worse verbal episodic memory performance was associated with lower OID scores only in the AD group. Free OID showed a broader and stronger pattern of associations with episodic memory and biomarkers compared with total OID. Threshold regression between free OID and Aβ42/40 identified a subthreshold value (<0.94) above the clinical cutoff (<0.86), capturing nine non-demented individuals within the gray zone between these cutoffs.

**Conclusions:**

Our findings support an association between amyloid levels and olfactory performance in the AD spectrum, underscoring the potential of smell tests as cost-effective tools in multimodal stratification frameworks. In dementia stages, medial temporal atrophy accompanied by memory impairment may be the main correlate of olfactory deficits.

**Supplementary Information:**

The online version contains supplementary material available at 10.1186/s13195-026-02073-w.

## Introduction

The progression of Alzheimer’s disease (AD) has been associated with olfactory deficits, typically assessed using odor identification (OID) standardized tests [1–4]. Olfactory decline has been suggested to precede cognitive decline [5, 6], likely reflecting early pathology and atrophy in the olfactory brain circuit, particularly in medial temporal lobe (MTL) structures [7–10]. In this context, examining the associations between AD biomarkers and OID at early disease stages is crucial. Such research could help clarify the role of olfactory deficits as potential early indicators of AD pathology and support the use of olfactory testing for risk stratification.

Early olfactory deficits may be partially driven by the shared mechanisms related to the deterioration of declarative memory later in the AD spectrum, as suggested by a reported positive association between episodic memory and OID in individuals with subjective cognitive complaints and no evident cognitive impairment [11]. Similar relations have been reported in aging studies [12]. In the context of AD development, this relationship may reflect sequential neurodegenerative processes that affect OID and episodic memory at different stages [5, 6].

Prior research on the association between AD-related protein biomarkers (measured by cerebrospinal fluid [CSF] and positron emission tomography [PET]) and OID performance supports the link between higher levels of both amyloid and tau pathology and poorer OID in individuals with mild cognitive impairment (MCI) and AD dementia [13–18]. Specifically, Klein et al. analyzed pooled data from 41 cognitively unimpaired, MCI, and AD individuals from different cohorts, showing that a higher tau uptake in the medial temporal cortex, hippocampus, middle and inferior temporal gyri, inferior parietal cortex, and posterior cingulate was associated with poorer OID [16]. Wang et al. further reported that higher amyloid uptake in the posterior cingulate cortex/precuneus, frontal, parietal, and lateral temporal cortices was associated with poorer OID in a pooled clinical sample of 164 patients, including cognitively unimpaired, MCI, and AD cases [17]. Overall, many of these studies lack stratified analyses by diagnostic group. This gap limits our understanding of how the association between AD proteinopathy and olfaction evolves across the clinical continuum [14–18].

Magnetic resonance imaging (MRI) studies have pinpointed the relationship between regional MTL atrophy (e.g., in the amygdala, entorhinal cortex, hippocampus, and parahippocampal gyrus) and poorer OID performance in MCI and AD dementia [11, 19–26]. On the other hand, vascular markers have been less explored, which represents another gap in the literature. Cerebrovascular disease co-pathology may be associated with the deterioration of OID across the clinical continuum, as suggested by findings on the association between white matter lesions and poorer OID performance in MCI [24]. We recently reported an association between a higher white matter hyperintensities (WMH) volume and faster OID decline in aging [27]. Previous studies have suggested that atherosclerosis in the supplying blood vessels to olfactory‑related regions may be a vascular mechanism contributing to olfactory dysfunction [28, 29].

Subjective cognitive impairment (SCI) has emerged as a group at risk of progression to MCI and AD dementia [30–32]. This group at risk presents self-reported cognitive impairment along with normal performance in standardized neuropsychological tests. A recent meta-analysis provided evidence that SCI individuals exhibit lower OID performance compared to controls [33], suggesting potential early dementia‑related changes before objective cognitive impairment becomes detectable. Nevertheless, there is a notable gap in the literature regarding how dementia biomarkers and cognitive measures are associated with OID among individuals with SCI. Only one PET study addressed the relationship between regional tau uptake and OID performance, analyzing seven SCI individuals pooled with 14 cognitively normal individuals [34]. The results showed that greater tau deposition in the entorhinal cortex, fusiform, inferior gyri, and parahippocampal gyri was associated with poorer OID [34]. In a subset of individuals with available amyloid‑PET, no associations were found between regional amyloid uptake and OID [34]. Additionally, previous studies reported associations between lower hippocampal volume and thinner entorhinal cortex with poorer OID in SCI [11, 35].

Both SCI and MCI typically occur along a clinical continuum associated with AD dementia, representing groups at risk [30–32, 36]. Many of these individuals do not progress to a dementia stage. Therefore, there is an urgent need for risk stratification. In this context, OID testing has shown promising results in identifying individuals with higher progression to dementia [1, 37]. Recent evidence has examined whether the interaction between subjective memory complaints and subjective olfactory impairment relates to incident AD dementia, reporting a trend toward increased risk [38].

Overall, the available evidence supports relationships between AD‑related pathology, neurodegeneration, and poorer OID ability across the clinical spectrum. Two key research questions remain largely unanswered: (1) What is the relationship between AD-related biomarkers and OID performance in early at-risk clinical groups, particularly in individuals without evident cognitive impairment? and (2) Could cerebrovascular pathology be associated with OID deficits across the clinical AD continuum?

Based on previous evidence, we hypothesized that in individuals with SCI, who typically show minimal atrophy, poorer OID performance would be primarily associated with early AD-related changes, particularly amyloid pathology. In contrast, in individuals with cognitive impairment (MCI and AD dementia), we expected poorer performance to be more strongly associated with MTL neurodegeneration.

Addressing these research questions and hypotheses is essential for understanding the potential biological underpinnings of poorer OID ability in individuals at risk of progression to AD dementia, particularly those without evident cognitive impairment. Such insights may inform future patient selection strategies for early interventions and clinical trials. To this end, we investigated the associations between a set of biomarkers of AD pathology, neurodegeneration, and cerebrovascular burden and OID performance in a naturalistic clinical cohort of individuals classified as SCI and diagnosed with MCI and AD dementia.

## Methods

### Participants

The study sample consisted of patients consecutively recruited at the Karolinska University Hospital Medical Unit Aging Memory Clinic in Solna, Sweden, between September 2021 and October 2023. In this specialized outpatient clinic, individuals with cognitive complaints are referred by general practitioners in primary and occupational health care in the catchment area (northern Stockholm) and the entire Stockholm region if they are under 65 years of age, or up to 70 years old if they are still working. The medical evaluation before referral encompasses anamnesis, MRI or computerized tomography scan, and Mini-Mental State Examination (MMSE). Around one month after referral, each patient is evaluated within one week, which results in a consensus diagnosis based on all test results, including biomarkers [39]. The participants of this study underwent olfactory testing in addition to the routine examination at the clinic. Olfactory performance was not considered for the subsequent diagnosis.

The harmonized dementia examination at the memory clinic follows the Swedish Board of Health and Welfare guidelines, including comprehensive medical and neurologic examination, medical and informant-based history, neuropsychological evaluation, blood chemistry, MRI acquisition, *APOE* genotyping, and CSF biomarker analysis. A multidisciplinary team (physicians, neuropsychologists, occupational therapist, speech and language pathologist, nurses, and physical therapist) set a consensus diagnosis for each evaluated patient based on the Diagnostic and Statistical Manual of Mental Disorders, Fifth Edition (DSM-5) criteria and coded according to the International Classification of Diseases, Tenth Revision (ICD-10) [39].

During the study period, 303 patients were enrolled. For the current study, we selected patients from three main diagnostic groups: SCI, MCI, and AD dementia. Patients are classified as SCI when they report cognitive complaints and do not meet the clinical criteria for MCI or dementia [39]. After excluding 6 patients with Parkinson’s disease, 16 patients with other diagnoses (2 alcohol-related dementia, 1 delirium, 1 other amnesia, 4 Pick’s disease, 6 unspecified dementia, 2 vascular dementia cases), 3 anosmic patients who reported a subjective loss of taste or smell after confirmed or suspected COVID-19 infection (2 SCI, 1 MCI), and 45 patients without available CSF data, 233 patients were included in the analyses. Our final study population consisted of 152 SCI, 50 MCI, and 31 AD patients with available olfactory and CSF data. MRI data were available for a subset of 99 SCI, 35 MCI, and 17 AD patients.

### Olfactory assessment

Olfactory performance was assessed with the Sniffin’ Sticks OID test, a normative test with high test-retest reliability [40, 41]. Typically, studies assessing smell identification rely exclusively on cued identification tasks. As a novelty, we also included free OID, a measure considered more cognitively demanding [42, 43]. Free OID is challenging even for healthy individuals and has been comparatively understudied for this reason. In contrast to cued OID, which has been linked more strongly to perceptual speed, free OID appears to engage in broader verbal and memory abilities [43]. This stronger reliance on memory abilities may make free OID potentially more sensitive to early AD‑related changes [43]. The neuropsychologist asked the patients to freely identify 16 common odors presented one by one in felt-tip pens. If the patient was unable to correctly identify a specific odor, a multiple forced-choice response was required, consisting of the selection of the label that best matched the odor within four written alternatives (one target and three distractors). One point was assigned for each correct identification. In cases of missing response on cued identification, due to e.g., an administrator mistake or allergy, the patients received 0.25 points (chance-level). The number of free or total (free or cued) identifications was used in the analyses. Both the free and total OID scores range from 0 to 16. We classified individuals as OD for descriptive purposes based on total OID score under the 25th percentile according to normative data [44], as follows: <13 (31–40 years old), < 12 (41–60 years old), < 11 (61–70 years old), and < 10 (71–80 years old).

### Neuropsychological assessment

The neuropsychological assessment addressed global cognition (Montreal Cognitive Assessment [MoCA]) [45] and specific cognitive domains. The majority of patients performed tests of verbal memory (Rey Auditory Verbal Learning Test [RAVLT]) [46], visuospatial abilities (The Rey-Osterrieth complex figure [ROCF]) [47], and perceptual speed/attention (Wechsler Adult Intelligence Scale-IV [WAIS-IV] Coding) [48]. We characterized global cognition in the sample with the MoCA. Total and delayed recall of the RAVLT (RAVLT-total and RAVLT-delayed) and WAIS-IV Coding were used for analyses focused on the relation between cognitive and OID performance.

### CSF collection and analysis

CSF was collected in sterile polypropylene tubes (Medicarrier art nr 67741) [39]. Amyloid-β 42 (Aβ42), amyloid-β 40 (Aβ40), phosphorylated tau 181 (p-tau181), total tau (t-tau), and neurofilament light chain (NfL) were measured with the Lumipulse G-series (Fujirebio Europe) fully automated chemiluminescent enzyme immunoassay at Karolinska University Hospital Laboratory [39]. Aβ42/40 ratio was computed and used as an amyloid marker in this study. In this study, we focused on the Aβ42/40 ratio, p-tau181, and NfL to capture distinct processes potentially linked to olfactory dysfunction. Of note, a lower Aβ42/40 ratio is associated with a higher Aβ accumulation in the brain. Aβ-positivity classification of patients (Aβ + vs. Aβ-) based on Aβ42/40 ratio (Aβ42/Aβ40[×10] < 0.86), further used in stratified analyses, was based on a previously established data-driven cutoff determined by Gaussian mixture modeling (GMM) [39].

### MRI acquisition and preprocessing

Brain images were acquired on a GE Discovery MR750 3T magnet (67 participants scanned up to September 2022) and a GE SIGNA Premier 3T magnet (84 participants scanned from September 2022; GE HealthCare, Milwaukee, WI, USA) according to routine protocols, including T1-weighted 3D BRAVO (Discovery MR750 and SIGNA Premier) or T1-weighted 3D MPRAGE (SIGNA Premier), and T2-weighted FLAIR 3D CUBE sequences.

Regional cortical and subcortical brain volumes were obtained by a multi-atlas segmentation method on 3D T1-weighted images (102 cortical and 31 subcortical regions) [49], and WMH volume (hereafter referred to as WM-hyper volume) as a proxy of cerebrovascular burden, was obtained on FLAIR images using the cNeuro cMRI software (Combinostics Oy, Finland), a fully automated brain MRI quantification used in the clinical setting. We selected the bilateral entorhinal cortex, amygdala, hippocampus, and parahippocampal gyrus as a priori regions of interest. These MTL structures are expected to show early atrophy across the AD course [9]. Moreover, the entorhinal cortex and amygdala are part of the so-called primary olfactory cortex, as these brain regions are one synapse away from the olfactory bulb [50]. The hippocampus and parahippocampal gyrus are part of the secondary olfactory cortex, receiving inputs from the primary olfactory cortex [50]. We adjusted the regional volumes for total brain tissue volume (TBTV) using a residual approach [51].

### *APOE* genotyping

*APOE* status was dichotomized as carriers (at least one allele) vs. non-carriers of the ε4 allele. *APOE* status was included as an additional covariate in subsequent analyses to assess its influence on the results. This was motivated by prior evidence showing that *APOE* ε4 carriership is associated with olfactory deficits, and that these deficits are linked to cognitive decline and the progression of AD dementia, even in cognitively normal individuals (see Murphy et al., 2018, for review [5]).

### Statistical analyses

Mean differences between diagnostic groups in demographic and clinical data were assessed using one-way analysis of variance (ANOVA; for continuous variables) or Kruskal-Wallis non-parametric ANOVA (for discrete variables, and for continuous variables when parametric assumptions were not met), followed by Least Significant Difference (LSD) and Dunn’s post-hoc pairwise comparisons, respectively. Effect sizes reflect Eta squared [η^2^] for one-way ANOVA and rank η^2^ for Kruskal-Wallis. For comparison between two groups, we used an independent Student’s *t*-test and Welch’s *t*-test (when homogeneity of variance was not met) for continuous variables and Mann–Whitney *U* test for discrete variables (and when parametric assumptions were not met), reporting effect sizes with Cohen’s *d* and rank biserial correlation, as appropriate. Frequency differences between groups in demographic and clinical data were assessed using Pearson’s chi-squared test, followed by post-hoc pairwise comparisons.

We examined one-tailed partial correlations (Spearman’s rho correlations adjusted for age, sex, and years of education [main model], and additionally adjusting for *APOE* status [supplementary model] to evaluate its potential influence on the observed associations) of OID performance with cognitive scores, CSF biomarkers, and MRI volumes for the whole sample and each of the three diagnostic groups. We applied one-tailed correlation tests because our hypotheses were directional, based on previous evidence [10, 12, 52], and grounded in the AD framework [5]: greater cognitive impairment and greater pathology would be associated with lower OID performance. We used Spearman’s rho partial correlations throughout the analyses because some key variables (e.g., CSF biomarkers and WM-hyper volume) were not normally distributed, sample sizes were relatively small in certain subgroups, and a non-parametric approach ensured comparability across analyses while remaining robust to capture both linear and monotonic relationships. We repeated these correlation analyses in the group of non-demented individuals (pooling SCI and MCI) and in the whole sample (pooling SCI, MCI, and AD dementia) based on Aβ-positivity (Aβ + vs. Aβ-). For these sets of correlation analyses across the study, we applied family-wise Bonferroni corrections (group by variable type [cognitive scores, CSF biomarkers, and MRI volumes] for each OID score) to adjust significance for multiple comparisons. We also reported significant associations at an uncorrected significance threshold of *p* < 0.05. No adjustments for multiple comparisons were applied in the subsequent analyses, as these were conducted based on the previous correlation analyses results.

We further explored the relationships of CSF and MRI markers with OID performance in the whole sample, addressing different proteinopathy pathways or pathogenic processes simultaneously. These analyses were conducted to determine which ones may be mainly associated with OID performance in the clinical spectrum. We classified the study markers into A (amyloid), T (tau), N (neurodegeneration), and V (vascular brain injury), with the specific markers being A = Aβ42/40 ratio, T = p-tau181, N = NfL and MTL volumes, and V = WM-hyper. We computed generalized additive models (GAMs). The models included CSF biomarkers and MRI volumes statistically significantly associated with OID performance in the main analyses for the whole sample (selecting the one with the highest correlation coefficient for N, if required) as smooth terms — functions that allow for capturing potential non-linear relationships. The smoothing parameters were estimated using restricted maximum likelihood (REML). Sociodemographic factors (main model) and additionally *APOE* status (supplementary model) were included as linear parametric terms.

Complementary, we aimed to examine whether threshold values could be identified in CSF biomarkers or MTL volumes that were associated with OID based on the results from GAMs, specifically where their association with OID showed an abrupt change after a specific value. Importantly, these analyses do not yield a threshold for OID performance but instead identify potential inflection points in biomarkers. To this end, we implemented threshold regression analyses. Step-type threshold regressions were fitted, and bootstrap resampling (1,000 samples) was employed to estimate 95% confidence intervals (CIs) for the threshold parameter. This approach has been previously used in a recent study examining biomarker thresholds for cognitive outcomes [53], showing its potential utility in detecting clinically relevant inflection points.

We conducted all descriptive analyses using JASP (version 0.96.0, JASP Team, Amsterdam, The Netherlands) [54]. All the other statistical analyses were performed using R (version 4.5.3, R Core Team, Vienna, Austria) [55] and the integrated development environment RStudio (version 2026.01.2, Posit Team, Boston, MA, US) [56], using the ‘ppcor’ package (version 1.1) for correlation analyses [57], the ‘mgcv’ (version 1.9.4) package for GAMs [58], and the ‘chngpt’ (version 2024.11.15) package for threshold regression analyses [59, 60]. All plots were created using the ‘ggplot2’ package (version 4.0.2) [61], and plots related to GAMs were generated using the ‘gratia’ package (version 0.11.2) [62, 63].

The number of missing data in each variable for the whole sample and the MRI subset is reported in Supplementary Table 1.

## Results

### Sample characteristics

The mean age of the study participants was 59.2 (*SD* = 5.6, range: 39–72), and 60.1% were females. Descriptive information by diagnostic group and for the whole sample can be found in Table 1. The MCI group was significantly older than the SCI group. The AD group had a significantly higher prevalence of *APOE* ε4 carriers than the SCI group.

**Fig. 1:**
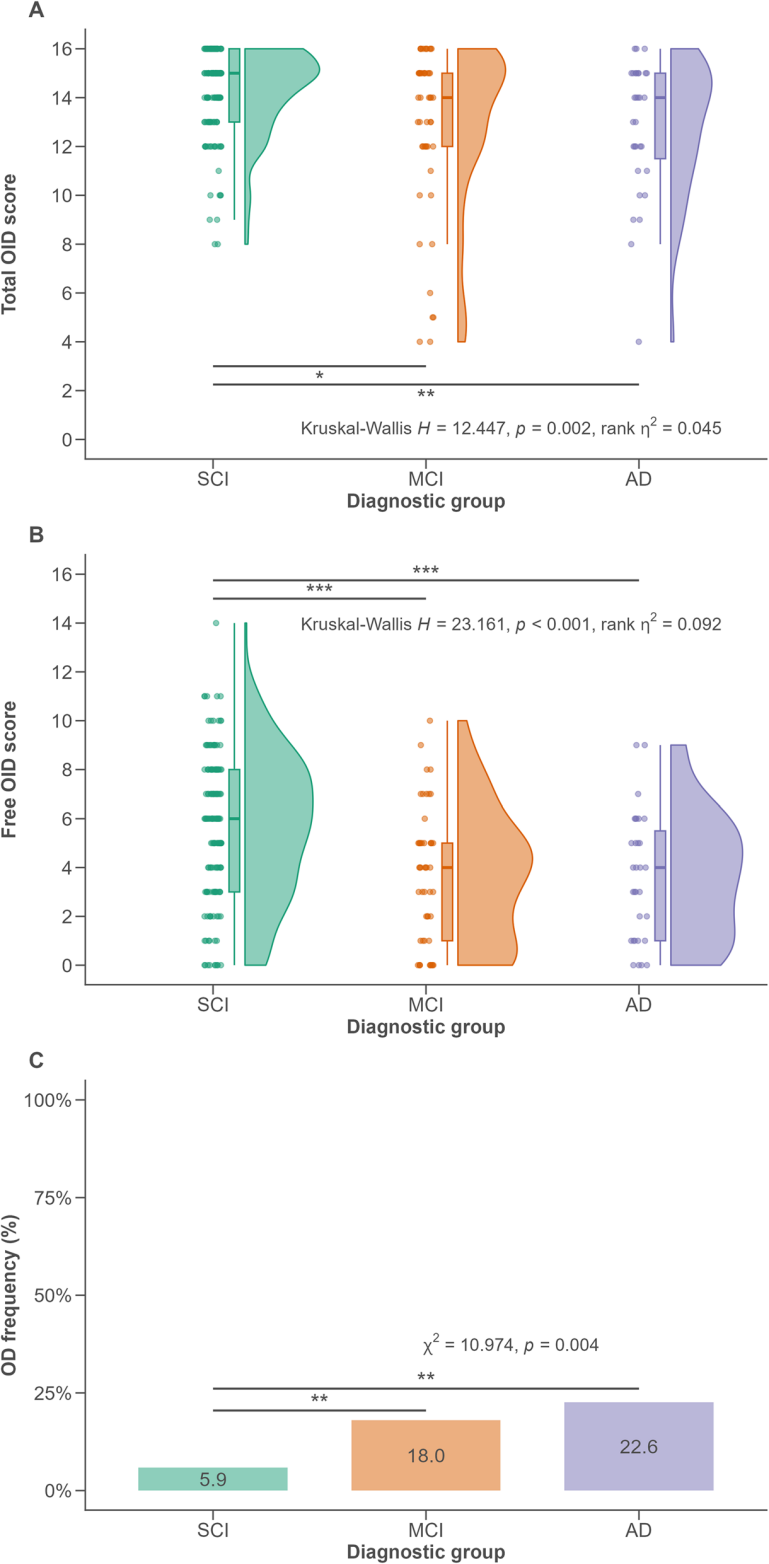
Olfactory performance by diagnostic group. Panel (**A**) Raincloud plots of total OID score by diagnostic group. Panel (**B**) Raincloud plots of free OID score by diagnostic group. Panel (**C**) Bar graph showing the prevalence of OD within the diagnostic groups. ^*^p < 0.05, ^**^p < 0.01, ^***^p < 0.001. *Abbreviations*: *AD* Alzheimer’s disease, *MCI* mild cognitive impairment, *OD* olfactory dysfunction, *OID* odor identification, *SCI* subjective cognitive impairment

Regarding olfactory performance, the MCI and AD groups performed significantly lower in total and free OID compared with the SCI group (Fig. 1, panels A & B), and had a higher prevalence of OD, from 5.9% in SCI to 18% in MCI and 22.6% in AD (Fig. 1, panel C).

**Table 1:** Demographic, clinical, neuropsychological, CSF, and MRI data for the whole sample and by diagnostic group

	All (*n* = 233)	SCI (*n* = 152)	MCI (*n* = 50)	AD (*n* = 31)	Test stat	*p*	Effect size
Age, years	59.2 (5.6)	58.5 (5.7)	61.5 (4.9)	58.7 (5.0)	5.690^F^	0.004^a^	0.047
Sex, female *n* (%)	140 (60.1)	99 (65.1)	24 (48.0)	17 (54.8)	5.015^χ2^	0.081	
Years of education	14.2 (3.1)	14.4 (3.1)	13.9 (3.2)	13.9 (3.2)	0.690^*F*^	0.502	0.006
*APOE* ε4 carrier, *n* (%)	106 (45.5)	62 (40.8)	24 (48.0)	20 (64.5)	6.565^χ2^	0.038^b^	
Olfaction							
Total OID score	15 (4–16)	15 (8–16)	14 (4–16)	14 (4–16)	12.447^*H*^	0.002^a, b^	0.045
OD, *n* (%)	25 (10.7)	9 (5.9)	9 (18.0)	7 (22.6)	10.974^χ2^	0.004^a, b^	
Free OID score	5 (0–14)	6 (0–14)	4 (0–10)	4 (0–9)	23.161^*H*^	< 0.001^a, b^	0.092
Cognitive scores							
MoCA	25 (9–30)	26 (17–30)	23 (13–30)	20 (9–26)	70.335 ^*H*^	< 0.001^a, b, c^	0.302
RAVLT-total	47 (10–71)	51 (18–71)	34 (12–60)	31.5 (10–54)	93.678 ^*H*^	< 0.001^a, b^	0.419
RAVLT-delayed	10 (0–15)	12 (1–15)	5 (0–15)	3 (0–11)	93.201^*H*^	< 0.001^a, b^	0.420
WAIS-IV Coding	52 (4–93)	57 (14–93)	46 (17–82)	58 (4–38)	35.951	< 0.001^a, b^	0.161
CSF markers							
Aβ42/40 ratio	0.99 (0.30–1.35)	1.03 (0.40–1.35)	0.76 (0.32–1.18)	0.54 (0.30– 0.75)	73.211^*H*^	< 0.001^a, b, c^	0.312
Positivity, *n* (%)	92 (39.5)	35 (23.0)	26 (52.0)	31 (100)	66.956^χ2^	< 0.001^a, b, c^	
p-tau181, pg/mL	39 (14–370)	34 (16–140)	47 (14–170)	99 (52–370)	66.291^*H*^	< 0.001^a, b,c^	0.281
NfL, pg/mL	710 (150–2280)	670 (250–2280)	900 (150–2090)	1090 (590–1910)	40.350 ^*H*^	< 0.001^a, b, c^	0.170
MRI volumes, mL^*^							
AMYG	2.21 (0.21)	2.25 (0.17)	2.17 (0.24)	2.01 (0.25)	11.534^*F*^	< 0.001^b, c^	0.135
ENT	4.54 (0.53)	4.60 (0.48)	4.52 (0.61)	4.23 (0.58)	3.589 ^*F*^	0.030^b^	0.046
HIP	7.30 (4.76–8.73)	7.47 (5.22–8.73)	7.03 (5.78–8.23)	6.45 (4.76–8.48)	22.908^*H*^	< 0.001^a, b^	0.141
PHIP	6.10 (0.60)	6.17 (0.59)	6.08 (0.62)	5.73 (0.49)	4.025^*F*^	0.020^b^	0.052
WM-hyper	1.39 (0.00–52.94)	1.09 (0.00–48.75)	3.20 (0.00–52.94)	2.08 (0.04–28.52)	13.762^*H*^	0.001^a^	0.079

Mean (standard deviation) (continuous variables) and median (minimum–maximum) (discrete variables, and for continuous variables when parametric assumptions were not met) are shown. Effect sizes correspond to Eta squared (η^2^) for one-way ANOVA and rank η2 for Kruskal-Wallis. ^a^Significant difference between SCI and MCI (*p* < 0.05); ^b^Significant difference between SCI and AD (*p* < 0.05); ^c^Significant difference between MCI and AD (*p* < 0.05); ^*F*^one-way ANOVA was used, ^*H*^Kruskal-Wallis non-parametric ANOVA was used, ^χ2^Pearson’s chi-squared test was used; ^*^MRI data was available for a subset of SCI (*n* = 99), MCI (*n* = 35), and AD (*n* = 17) patients

*Abbreviations*: *Aβ *amyloid-β, *Aβ42/40* amyloid-β 42/amyloid-β 40, *AD* Alzheimer’s disease, *AMYG* amygdala, *APOE* apolipoprotein E, *CSF* cerebrospinal fluid, *ENT* entorhinal cortex, *HIP* hippocampal, *MCI* mild cognitive impairment, *MoCA* Montreal Cognitive Assessment, *MRI* magnetic resonance imaging, *NfL* neurofilament light chain, *OD* olfactory dysfunction, *OID* odor identification, *PHIP* parahippocampal gyrus, *p-tau181* phosphorylated tau 181, *RAVLT* Rey Auditory Verbal Learning Test, *SCI* subjective cognitive impairment, *WAIS* Wechsler Adult Intelligence Scale, *WM-hyper* white matter hyperintensities

As expected, the AD group showed significantly lower global cognition (MoCA) in comparison to the SCI and MCI groups, with the MCI group also showing lower global cognition relative to the SCI group. The AD and MCI groups performed significantly lower in verbal episodic memory and perceptual speed than the SCI group. The AD group also had a significantly lower Aβ42/40 ratio, a higher prevalence of Aβ-positivity, and higher levels of p-tau181 and NfL compared with the SCI and MCI groups. There were significant differences in the same direction between MCI and SCI. Concerning MRI volumes, the AD group had significantly smaller bilateral volume in the amygdala, relative to the SCI and MCI groups, and in the entorhinal cortex and parahippocampal gyrus, relative to the SCI group. The AD and MCI groups had a smaller hippocampal volume than the SCI group. The MCI group had a significantly higher volume WM-hyper volume relative to the SCI group.

### Associations between cognitive and olfactory performance

Table 2 summarizes the associations of cognitive performance, CSF biomarkers, and MRI volumes with OID performance in the whole sample and across diagnostic groups.

**Table 2:** Associations of cognitive scores, CSF markers and MRI volumes with OID scores for the whole sample and stratified by diagnostic group

	All		SCI		MCI		AD	
	Total OID	Free OID	Total OID	Free OID	Total OID	Free OID	Total OID	Free OID
Cognitive scores								
RAVLT-total	0.085	0.240^*^	-0.079	0.078	0.168	0.127	0.313	0.406^†^
RAVLT-delayed	0.122^†^	0.248^*^	-0.003	0.119	0.147	0.078	0.248	0.472^*^
WAIS-IV Coding	0.227^*^	0.165^*^	0.092	0.002	0.310^†^	0.187	0.413^†^	0.229
CSF markers								
Aβ42/40 ratio	0.135^†^	0.259^*^	0.131	0.195^*^	-0.196	-0.007	0.415^*^	0.102
p-tau181	-0.077	-0.162^*^	-0.020	-0.013	0.114	-0.135	-0.112	-0.051
NfL	-0.101	-0.185^*^	0.032	-0.045	-0.127	-0.290^†^	-0.130	-0.296
MRI volumes								
AMYG	0.080	0.207^**^	-0.063	0.038	0.158	0.257	0.050	0.304
ENT	0.077	0.079	-0.073	-0.007	0.334^†^	0.306^†^	-0.173	-0.069
HIP	0.220^**^	0.244^**^	0.129	0.080	0.322^†^	0.230	0.518^†^	0.643^**^
PHIP	0.052	0.040	-0.025	-0.027	0.085	0.066	0.157	0.040
WM-hyper	-0.085	-0.058	0.120	0.003	-0.496^**^	-0.148	-0.190	-0.286

Spearman’s rho correlation coefficients are shown. ^†^*p* < 0.05 (uncorrected significance threshold), ^*^p < 0.0167, ^**^p < 0.010 (family-wise corrected significance thresholds)

*Abbreviations*: *Aβ *amyloid-β, *Aβ42/40* amyloid-β 42/amyloid-β 40, *AD* Alzheimer’s disease, *AMYG* amygdala, *CSF* cerebrospinal fluid, *ENT* entorhinal cortex, *HIP* hippocampal, *MCI* mild cognitive impairment, *MRI* magnetic resonance imaging, *NfL* neurofilament light chain, *OID* odor identification, *PHIP* parahippocampal gyrus, *p-tau181* phosphorylated tau 181, *SCI* subjective cognitive impairment, *WAIS* Wechsler Adult Intelligence Scale, *WM-hyper* white matter hyperintensities

In the whole sample, both RAVLT-total (ρ = 0.240, *p* = 1.648 × 10^− 4^) and RAVLT-delayed (ρ = 0.248, *p* = 1.120 × 10^− 4^) scores were positively associated with free OID score, while RAVLT-delayed score (ρ = 0.122, *p* = 0.036) was associated with total OID. WAIS-IV Coding scores were positively associated with both total OID (ρ = 0.227, *p* = 4.367 × 10^− 4^) and free OID (ρ = 0.165, *p* = 8.235 × 10^− 3^).

In the AD group, RAVLT-total (ρ = 0.406, medium effect, *p =* 0.034) and RAVLT-delayed were positively associated with free OID scores (ρ = 0.472, medium effect, *p* = 0.018). WAIS-IV Coding was positively associated with total OID in MCI (ρ = 0.310, medium effect, *p* = 2.154 × 10^− 2^) and AD (ρ = 0.413, medium effect, *p* = 3.514 × 10^ − 2^). There were no significant associations between verbal episodic memory and OID performance within the SCI and MCI groups.

### Associations between CSF biomarkers and olfactory performance

In the whole sample, lower Aβ42/40 ratio (ρ = 0.259, *p* = 3.746 × 10^− 5^), and higher p-tau181 (ρ=-0.162, *p* = 0.007) and NfL (ρ=-0.185, *p* = 0.003) levels were associated with lower free OID score. Lower Aβ42/40 ratio was also associated with lower total OID score (ρ = 0.135, *p* = 0.021).

**Fig. 2:**
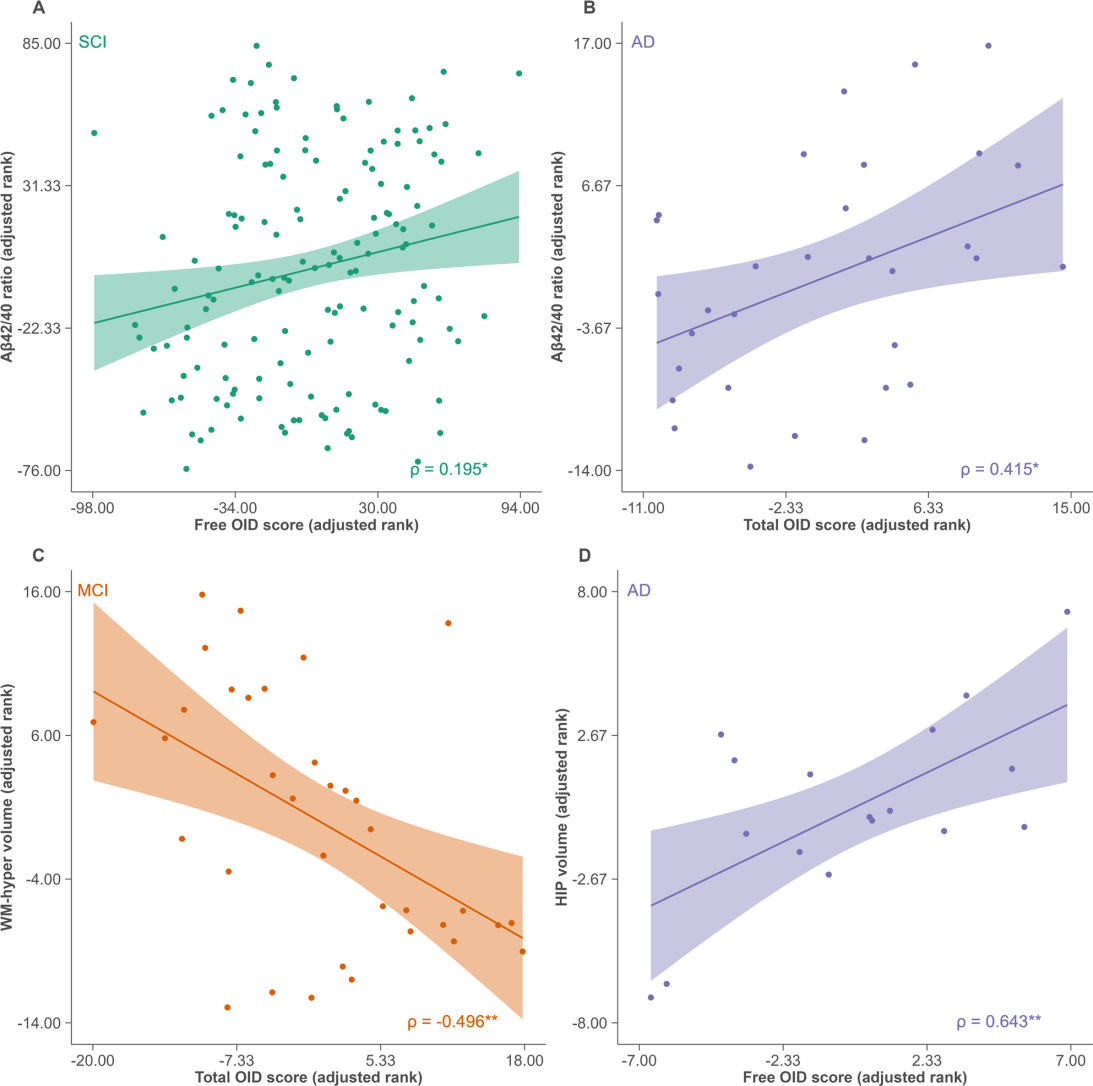
Significant associations of olfactory performance with CSF biomarkers and MRI volumes after multiple comparisons adjustment. Panel (**A**) Scatter plot depicting the association between free OID score and Aβ42/40 ratio in the SCI group. Panel (**B**) Scatter plot depicting the association between total OID score and Aβ42/40 ratio in the AD group. Panel (**C**) Scatter plot depicting the association between total OID score and WM-hyper volume in the MCI group. Panel (**D**) Scatter plot depicting the association between free OID score and HIP volume in the AD group. Adjusted ranks obtained from linear regression models with OID scores, CSF biomarkers, and MRI volumes as dependent variables and sex, age, and years of education as independent variables. Shaded areas around the regression lines represent 95% confidence intervals. ^*^p < 0.0167, ^**^p < 0.010. *Abbreviations*: *Aβ* amyloid-β, *Aβ42/40* ratio amyloid-β 42/amyloid-β 40 ratio, *AD* Alzheimer’s disease, *HIP* hippocampal, *MCI* mild cognitive impairment, *OID* odor identification, *SCI* subjective cognitive impairment, *WM-hyper* white matter hyperintensities

In the SCI group, a lower Aβ42/40 ratio was associated with a lower free OID score (ρ = 0.195, small effect, *p* = 0.009; Fig. 2, panel A), while in the AD group, a lower Aβ42/40 ratio was associated with a lower total OID score (ρ = 0.415, medium effect, *p* = 0.014; Fig. 2, panel B). In the MCI group, higher NfL levels were associated with lower free OID score (ρ=-0.290, small effect, *p* = 0.027).

### Associations between MRI volumes and olfactory performance

In the whole sample, a smaller hippocampal volume was associated with poorer performance in both total OID (ρ = 0.220, *p* = 0.004) and free OID (ρ = 0.244, *p* = 0.001), and a smaller amygdala volume was associated with poorer performance in free OID (ρ = 0.207, *p* = 0.006).

In the MCI group, a higher WM-hyper volume was associated with a lower total OID score (ρ=-0.496, medium effect, *p* = 0.002; Fig. 2, panel C). Additionally, some MTL volumes were associated with OID performance in the MCI group (medium effects), namely: entorhinal cortex (total OID: ρ = 0.334, *p* = 0.031; free OID: ρ = 0.306, *p* = 0.045) and hippocampal (total OID: ρ = 0.322, *p* = 0.036) volumes. Lastly, in the AD group, a smaller hippocampal volume was associated with a lower OID score (total OID, ρ = 0.518, large effect, *p* = 0.029; free OID: ρ = 0.643, large effect, *p* = 0.007; Fig. 2, panel D).

### Correlates of olfactory performance by Aβ-positivity

Descriptive information of non-demented individuals (pooled SCI and MCI group) by Aβ-positivity (Aβ-, *n* = 162; Aβ+, *n* = 38) can be found in Supplementary Table 2. Of note, non-demented Aβ + individuals performed significantly lower in free OID compared with non-demented Aβ- individuals.

Table 3 summarizes the correlates of OID performance in non-demented individuals, stratified by Aβ-positivity. In the non-demented Aβ + group, WM-hyper volume was negatively associated with total OID (ρ=-0.517, large effect, *p* = 0.004), amygdala volume was positively associated with total OID (ρ = 0.365, medium effect, *p* = 0.036), and entorhinal volume with free OID (ρ = 0.369, medium effect, *p* = 0.035). In the non-demented Aβ- group, the Aβ42/40 ratio was positively associated with free OID (ρ = 0.150, small effect, *p* = 0.030) and hippocampal volume with total OID (ρ = 0.205, small effect, *p* = 0.019). Moreover, RAVLT-total and free OID performance were positively associated in the non-demented Aβ + group (ρ = 0.405, medium effect, *p* = 9.743 × 10^− 4^).

**Table 3:** Associations of cognitive scores, CSF markers and MRI volumes with OID scores in non-demented Aβ-, non-demented Aβ + and all Aβ + groups

	Non-demented Aβ-		Non-demented Aβ+		All Aβ+	
	Total OID	Free OID	Total OID	Free OID	Total OID	Free OID
Cognitive scores						
RAVLT-total	-0.014	0.055	0.045	0.405^*^	0.256^*^	0.430^*^
RAVLT-delayed	0.047	0.133	0.074	0.224^†^	0.259^*^	0.318^*^
WAIS-IV Coding	0.153^†^	0.074	0.214	0.247^†^	0.371^*^	0.327^*^
CSF markers						
Aβ42/40	0.141	0.145^†^	0.015	0.141	0.141	0.156
p-tau181	0.021	0.044	0.039	-0.079	-0.124	-0.138
NfL	-0.056	-0.076	-0.034	-0.085	-0.164	-0.191^†^
MRI volumes						
AMYG	-0.065	-0.012	0.180	0.325^†^	0.197	0.336^**^
ENT	0.040	0.028	0.080	0.088	0.088	0.067
HIP	0.185^†^	0.144	0.211	0.136	0.241^†^	0.285^†^
PHIP	0.074	0.021	-0.146	-0.153	-0.057	-0.073
WM-hyper	0.095	-0.024	-0.365^**^	-0.074	-0.333^**^	-0.158

Non-demented Aβ- group includes SCI and MCI Aβ- individuals. Non-demented Aβ + group includes SCI and MCI Aβ + individuals. All Aβ + group includes SCI, MCI and AD Aβ + individuals. Amyloid-positivity was defined as Aβ42/Aβ40[×10] < 0.86. Spearman’s rho correlation coefficients are shown. ^†^*p* < 0.05 (uncorrected significance threshold), ^*^p < 0.0167, ^**^p < 0.010 (family-wise corrected significance thresholds)

*Abbreviations*: *Aβ *amyloid-β, *Aβ42/40* amyloid-β 42/amyloid-β 40, *AD* Alzheimer’s disease, *AMYG* amygdala, *APOE* apolipoprotein E, *CSF* cerebrospinal fluid, *ENT* entorhinal cortex, *HIP* hippocampal, *MCI* mild cognitive impairment, *MRI* magnetic resonance imaging, *NfL* neurofilament light chain, *PHIP* parahippocampal gyrus, *p-tau181* phosphorylated tau 181, *SCI* subjective cognitive impairment, *WAIS* Wechsler Adult Intelligence Scale, *WM-hyper* white matter hyperintensities

The associations with OID followed a similar pattern in the whole group of Aβ + individuals (pooling SCI, MCI and AD). Descriptive information is available in Supplementary Table 3.

### Associations of olfactory performance adjusting for *APOE* status

Results after additionally adjusting for *APOE* status are available in Supplementary Tables 4 and 5. Of note, the associations between Aβ42/40 ratio and OID were no longer significant in SCI and non-demented Aβ- individuals.

### Associations of CSF biomarkers and MRI volumes with olfactory performance adjusted for each other

GAMs revealed significant associations between olfactory performance and CSF/MRI markers in the whole sample after addressing different potential proteinopathy pathways and neurodegenerative processes simultaneously. In the model with total OID performance as an outcome, only hippocampal volume was significantly associated (*F* = 4.750, *p* = 0.031), whereas Aβ42/40 ratio did not reach significance (*F* = 1.103, *p* = 0.295; Table 4). This model explained 18.6% of the deviance, with an adjusted R² of 0.158. In the model with free OID performance as an outcome, significant associations were observed for Aβ42/40 ratio (*F* = 5.862, *p* = 0.017) and hippocampal volume (*F* = 5.476, *p* = 0.021); the association with ptau-181 was not significant (*F* = 0.138, *p* = 0.710) (Table 4). This model explained 33.7% of the deviance, with an adjusted R² of 0.309.

**Table 4:** Generalized additive models with OID performance as an outcome in the whole sample

	Estimate	Std. Error	Test stat.	*p*
Model: Total OID				
Parametric terms				
(Intercept)	20.546	2.367	8.262	7.68 × 10^− 15^
Age	-0.086	0.036	-2.415	0.017
Sex	-1.313	0.398	-3.301	0.001
Years of education	0.015	0.065	0.233	0.816
Smooth terms				
Aβ42/40 ratio	-	-	1.103	0.295
HIP volume	-	-	4.750	0.031
Model: Free OID				
Parametric terms				
(Intercept)	13.018	2.576	5.054	1.30 × 10^− 6^
Age	-0.102	0.039	-2.619	0.010
Sex	-2.342	0.437	-5.360	3.27 × 10^− 7^
Years of education	0.073	0.071	1.030	0.305
Smooth terms				
Aβ42/40 ratio	-	-	5.862	0.017
p-tau181	-	-	0.138	0.710
HIP volume	-	-	5.476	0.021

Parametric terms are reported with *t*-statistics, and smooth terms with *F*-statistics

*Abbreviations*: *Aβ *amyloid-β, *Aβ42/40* amyloid-β 42/amyloid-β 40, *HIP* hippocampal, *OID* olfactory identification, *p-tau181* phosphorylated tau 181

**Fig. 3:**
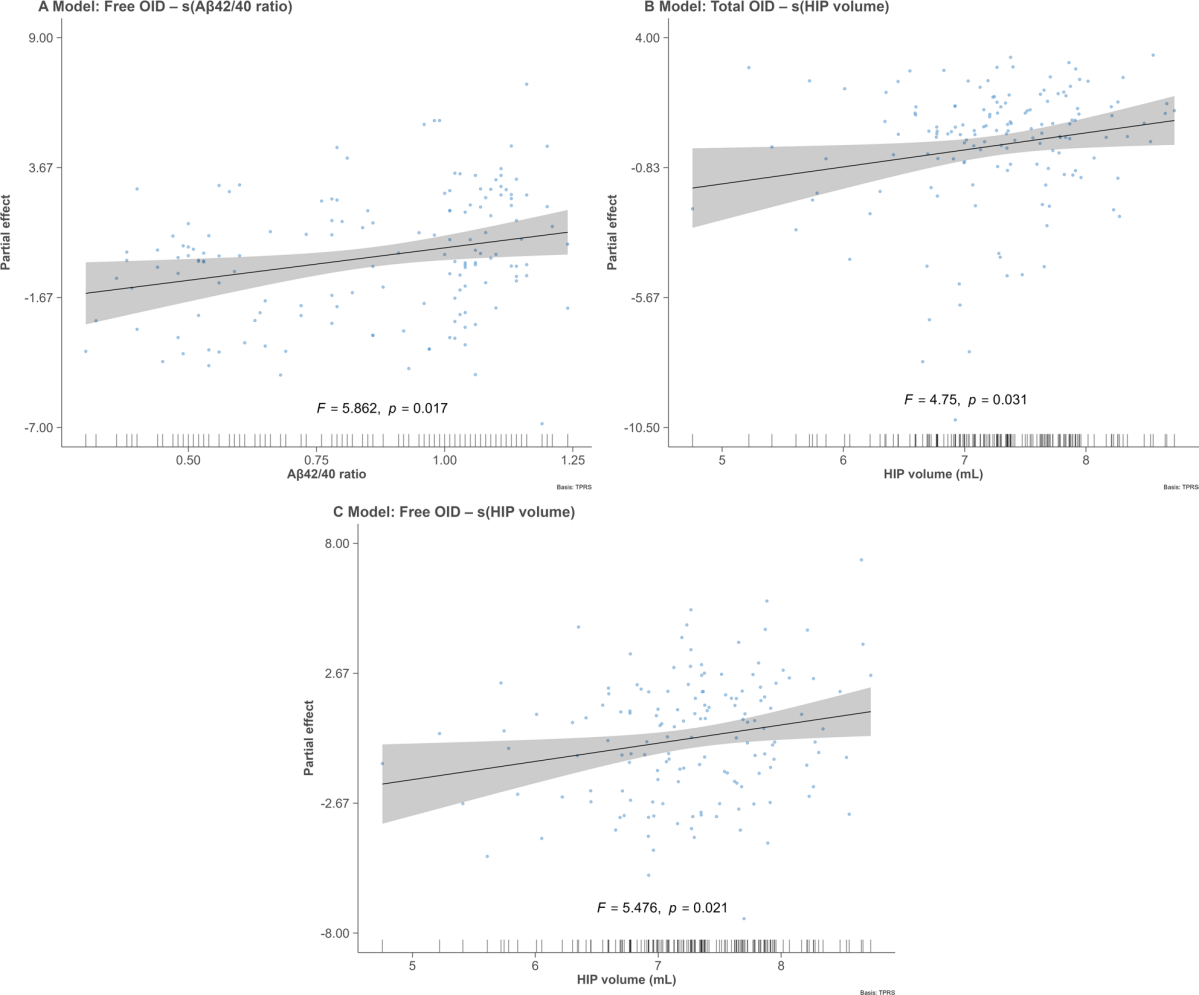
Significant partial effects of CSF and MRI biomarkers on OID performance based on generalized additive models. Panel (**A**) Partial effect of Aβ42/40 ratio on free OID score. Panel (**B**) Partial effect of HIP volume on total OID score. Panel (**C**) Partial effect of HIP volume on free OID score. Effects estimated using thin plate regression splines (TPRS) in a GAM model adjusted for sex, age, and years of education. The notation s(*x*) indicates a smooth term for variable *x*. Shaded areas represent 95% confidence intervals. *Abbreviations*: *Aβ* amyloid-β, *Aβ42/40* amyloid-β 42/amyloid-β 40, *HIP* hippocampal

Visual inspection of the partial effect plots indicated that the relationships between these specific markers (Aβ42/40 ratio and hippocampal volume) and OID performance were approximately linear (Fig. 3, panels A to C).

After adjustment for *APOE* status, only hippocampal volume remained significantly associated with free OID (Supplementary Table 6).

### Threshold regression analyses

The association between the Aβ42/40 ratio and free OID showed a significant stepwise change at the data‑driven threshold of 0.94 (95% CI: 0.61–1.10, *p* = 5.958 × 10^− 6^). Nine non-demented individuals (7 SCI and 2 MCI) fell within the gray zone between this threshold and the clinical cutoff (< 0.86). Notably, eight of them were *APOE* ε4 carriers (6 SCI and 2 MCI). These individuals exhibited low free OID performance, with a median score of 1 (range: 0–8). Although the estimated threshold remained unchanged after adjustment for *APOE* status, the stepwise effect was no longer statistically significant (0.94; 95% CI: 0.56–1.16, *p* = 0.203).

## Discussion

In this naturalistic memory clinic cohort spanning the clinical spectrum from SCI to MCI and AD dementia, we provide an integrated characterization of the cognitive and biological correlates of OID performance. Lower Aβ42/40 ratio and reduced hippocampal volume consistently emerged as the strongest correlates of poorer OID, underscoring the potential link between olfactory deficits and AD‑related amyloid and neurodegenerative processes. Notably, OID performance allowed us to identify a subgroup of non-demented individuals with a less favorable clinical profile despite exhibiting Aβ42/40 values above the established clinical cut‑off. This suggests that OID may capture an early vulnerability along the AD continuum and could complement established biomarkers in future risk‑stratification and early‑detection frameworks.

Smell deficits increased across diagnostic groups, with OD prevalence rising from approximately 6% in SCI to 18% in MCI, and approximately 23% in AD dementia. This observation aligns with existing evidence and likely reflects a gradual decline in olfactory abilities as AD progresses [5, 11, 33, 64]. The relatively low prevalence of OD in the present study may be due to our sample being relatively young and healthy. Notably, the free OID task, which is considered more cognitively demanding [42, 43], showed a larger effect size when assessing differences between groups. Free OID engages a broader range of cognitive resources [43] and may be particularly sensitive to subtle differences in cognitive abilities, potentially making it a more informative marker in healthier or high-functioning populations.

Verbal episodic memory showed a significant association with free OID in the whole sample, but this association reached significance only in AD dementia at the group level. This relationship likely reflects a major deficit in the ability to identify smells once AD dementia is diagnosed, with increasing cognitive deficits depending on both typicality and severity of the disease [65].

It is noteworthy that we included free OID, a measure proposed as cognitively more demanding and more dependent on verbal and memory processes, whose use has been largely restricted to research settings [42, 43]. In our sample, free OID showed a larger effect size in group comparisons. Additionally, it demonstrated a broader and generally stronger pattern of associations with underlying pathology, including amyloid burden in non-demented individuals and hippocampal atrophy in those with AD dementia. This task requires spontaneous odor naming, and recent findings suggest that it engages broader cognitive resources compared with cued OID [43]. As such, it may be more sensitive to subtle cognitive differences in populations with relatively preserved performance on standard neuropsychological tests. Of note, total OID, which also includes cued identification, showed a stronger correlation pattern with perceptual speed. This observation aligns with the notion that free OID may rely more heavily on verbal memory processes, whereas cued OID may rely more on perceptual speed [43]. These results suggest that incorporating free OID into clinical research may complement cued OID and enhance the detection of subtle cognitive changes that conventional neuropsychological assessments may miss.

When exploring the CSF markers, a lower Aβ42/40 ratio and a higher p-tau181 were associated with worse OID performance in the whole sample. Notably, the association with p-tau181 was no longer significant after adjustment for the Aβ42/40 ratio. This contrasts with previous evidence suggesting that tau pathology may be the main contributor to olfactory deficits, with attenuated or diluted amyloid effects when adjusting for tau, as shown in neuropathological and PET studies [66–68]. Differences from previous reports may partly reflect methodological and sample characteristics, including PET’s higher sensitivity, the younger age of our cohort, and its early clinical staging, which may have increased the likelihood of capturing individuals in amyloid-driven phases.

At the group level, only the Aβ42/40 ratio was linked with OID performance in SCI and AD. The same pattern was observed in the non-demented Aβ-negative group, which encompasses SCI and MCI. These results suggest that amyloid pathology may be associated with olfactory deficits from the earliest stages of AD, at subthreshold amyloid levels. One could hypothesize that the olfactory deficits observed in AD are due to amyloid pathology in early stages, before cognitive impairment is already evident [5, 6]. This is in line with olfactory deficits and decline being associated with dementia more than 10 years before diagnosis [67, 69, 70]. Furthermore, this early link between amyloid and olfaction might be particularly relevant for SCI individuals, as suggested by prior work exploring the interaction between subjective olfactory and memory complaints, which has reported a trend toward increased risk of incident AD dementia [38]. Of note, in our cohort, the associations with amyloid in individuals with SCI and in Aβ‑negative non‑demented individuals were largely driven by *APOE* ε4 carriers. The associations were attenuated and became non‑significant after adjusting for *APOE* status. This pattern is consistent with previous studies showing a higher prevalence of olfactory dysfunction in cognitively unimpaired *APOE* ε4 carriers, linked to transitions to MCI and AD dementia (see Murphy et al., 2019, for a review [5]). Notably, the earliest accumulation of amyloid impacts key structures for the sense of smell [71] and has been reported to affect brain functional connectivity, particularly within the default mode network (DMN) [72], which is crucial for cognition [73]. Recently, it has been described that olfactory brain networks may have privileged access to DMN compared to other senses [74]. Both networks and their interconnections may share a common vulnerability to amyloid levels at a very early AD stage. Therefore, amyloid pathology may act as an initial driver of the divergence of olfactory decline trajectories from normal aging in AD development, before significant contributions from other proteinopathy pathways or pathogenic processes.

While our results showed that the relationship between amyloid and olfactory function in the whole sample follows a linear trend, we detected an abrupt change in the association from a subthreshold in the Aβ42/40 ratio (Aβ42/40 < 0.94), which differs from the established Aβ-positivity cutoff (Aβ42/40 < 0.86). Notably, nine non‑demented individuals fell within the gray zone between these two thresholds, most of whom were *APOE* ε4 carriers, including six with SCI and two with MCI. This subthreshold cut‑off may reflect very early amyloid‑related alterations preceding conventional positivity thresholds, potentially bearing clinical relevance for predementia stages of AD, especially in *APOE* ε4 carriers [5].

Regarding markers related to neurodegeneration, higher NfL levels and smaller volumes of the amygdala and hippocampus were associated with worse OID ability in the whole sample. At the group level, hippocampal volume was positively associated with olfaction in MCI and AD, in line with previous reports [11, 19, 21–24, 26, 75]. In MCI, the associations with NfL and entorhinal cortex volume were also significant, but did not remain significant after adjusting for *APOE* status. The regionality of the findings and their relation with *APOE* ε4 carriership in MCI suggest that olfactory deficits may serve in part as a surrogate marker of AD-typicality [65], potentially reflecting a limbic-predominant atrophy pattern. This link between olfactory deficits and AD-typicality is reinforced by the observed positive association of hippocampal and amygdala volumes with olfactory performance in non-demented Aβ-positive individuals. These associations may reflect impairment of key processes involved in OID within the AD spectrum. Amygdala dysfunction could affect emotional evaluation of odors (e.g., salience, valence), while hippocampal damage may compromise memory-based identification [71].

A recently published study on a Canadian multi-center sample from the COMPASS-ND study reported a positive association between entorhinal cortex thickness and OID in individuals diagnosed with SCI and MCI [11]. In our study, a similar association between entorhinal cortex volume and OID was observed in the MCI group and in non-demented Aβ-positive individuals. At the macrostructural level, the entorhinal cortex may begin to atrophy later than the hippocampus, amygdala, and parahippocampal cortex [9]. These last regions may thus be more closely related to olfactory deficits early in AD progression. On the other hand, we did not find any significant association between brain volumes and olfactory performance in the SCI group. The differences in sample characteristics may partly explain the discrepancy with previous findings [11], particularly the older age of SCI individuals in the COMPASS-ND study (mean = 70.1, *SD* = 7.0), also in comparison with another study reporting a significant correlation with hippocampal volume in a clinical SCI group (mean = 67.3, *SD* = 5.7) [35]. The population of older individuals with subjective cognitive complaints is intrinsically heterogeneous, and the findings may reflect a mixture of aging-related heterogeneity and AD-related processes.

A cerebrovascular component of smell impairment has previously been proposed, based on observed associations with cardiovascular and cerebrovascular diseases, as well as related risk factors in aging [28, 76–80]. It has been hypothesized that reduced cerebral perfusion and hypoxia, resulting from atherosclerosis in the vessels supplying olfactory-related brain regions, may contribute to these sensory deficits [28, 29]. Regarding cerebrovascular burden in association with olfactory deficits in aging, the cross-sectional studies show mixed results, pointing to a negative or null association [27, 81–83]. A recent longitudinal study, however, found an association between a faster increase in the WM-hyper volume and accelerated OID decline [27]. The prevalence of cerebrovascular co-pathologies in AD and their relationship with cardiovascular risk factors and multimorbidity are increasingly acknowledged [84–86]. Nevertheless, the specific contribution of cerebrovascular burden to this sensory dysfunction across the AD spectrum remained underexplored. We found that a higher WM-hyper volume was associated with worse OID in individuals with MCI, which aligns with a previous report showing an association between severe deep white matter lesions and low olfactory scores in a clinical sample of MCI patients [24]. The association was replicated among non-demented individuals with Aβ-positive status, indicating that this observation does not appear specific to MCI and may be driven by amyloid-related processes. Further replication in all Aβ-positive individuals reinforces this interpretation. These findings suggest that cerebrovascular effects of β-amyloid, possibly related to cerebral amyloid angiopathy (CAA), may underlie the observed association [87]. Of note, white matter lesions have been linked to CAA [88]. Further work is needed to determine how regional patterns of these lesions may affect brain olfactory pathways.

Lastly, the relationships between key AD markers, namely amyloid levels and hippocampal volume, and OID followed a linear trajectory in our sample. This suggests a gradation between the level of pathology and olfactory deficits (OID worsening as pathology increases), consistent with their progressively higher prevalence from predementia stages to AD [5, 33, 64], and their long-term association with incident dementia [67, 69, 70]. Overall, our results pinpoint that different processes that impact the sense of smell may accumulate as layers over the course of the disease. The effect of amyloid appears early and remains relevant even in dementia stages, where additional pathological processes, such as more extensive neuronal loss within the olfactory brain circuit, also play an important role. Future studies using longitudinal data should address the hypothesized temporal contribution of these mechanisms to olfactory decline throughout the course of the disease, from amyloid and tau in earlier stages to MTL atrophy in later phases.

A major strength of this study is its use of routinely collected clinical data to investigate protein, brain, and cognitive correlates of smell function within a real-world memory clinic setting. In addition, we examined for the first time the association between fluid biomarkers and olfactory performance in a group of individuals with subjective cognitive complaints.

Our study lacks PET imaging to capture early protein deposition in the olfactory brain circuit [34, 68, 89]. In future studies, the use of more sensitive structural imaging markers, such as gray matter mean diffusivity in olfactory regions [90], may help detect microstructural features associated with smell impairment and partially overcome this limitation. The single-clinic setting and specific diagnostic workflow might limit the generalizability of our findings. The relatively young age of the cohort limits the generalizability of the findings to late‑onset AD. This age distribution reflects the specific referral patterns for the current memory clinic [91]. Additionally, we lack a reference group without cognitive complaints. We did not investigate Lewy body (LB) pathology, which could be a primary driver of olfactory deficits in MCI-LB cases [92, 93], or contribute to a further impairment of the olfactory network in individuals with mixed pathology [94]. Finally, clinical and research settings should incorporate domain‑specific assessments of cognitive complaints, as subjective memory complaints appear to be more strongly related to the risk of AD dementia [38]. Preliminary evidence examining the interaction between subjective memory and olfactory complaints suggests that their co‑occurrence may identify individuals at increased risk of AD dementia [38].

## Conclusion

Our findings suggest that amyloid pathology may contribute to olfactory deficits from the earliest stages of the AD continuum. Importantly, OID performance enabled us to identify a subgroup of non-demented individuals with subthreshold Aβ42/40 levels who nevertheless exhibited a potentially less favorable clinical and biological profile, suggesting that olfactory testing may help flag early risk even before conventional biomarker thresholds are crossed. In the future, combining olfactory markers with AD‑related fluid biomarkers may support earlier and more refined stratification of individuals at increased risk of cognitive decline. Our results also indicate that cerebrovascular burden related to amyloid pathology may further contribute to smell impairment. In later clinical stages, MTL atrophy may also contribute to worse odor OID. Altogether, these findings highlight the need for further research on the use of olfactory testing in multimodal assessment strategies aimed at early detection and risk stratification in memory clinics and clinical trials.

## Supplementary Information

Below is the link to the electronic supplementary material.

Supplementary Material 1.

## Data Availability

The research team is open to requests for data collected in this study. Study plan (including the research question, planned analysis, and data required) will be evaluated on a case-by-case basis. Shared data will encompass the data dictionary and de-identified data only. Analysis will be conducted in collaboration with the research team. Access is subject to the GEDOC legal framework. An access agreement will be prepared and signed by both parties.
