# Supplementary material for: Alzheimer’s disease and cerebrovascular biomarkers in relation to odor identification in a naturalistic clinical cohort
Source: Alzheimers Res Ther. 2026 Jun 3;18:138. doi: 10.1186/s13195-026-02073-w (PMC13244850; doi:10.1186/s13195-026-02073-w)
Supplement: Supplementary file 1 — Supplementary Material 1. [file 13195_2026_2073_MOESM1_ESM.docx]

**Supplementary Table 1** Number of missing values by diagnostic group for each variable in the whole sample and the MRI subset

|  | | | | **SCI** | **MCI** | **AD** |
| --- | --- | --- | --- | --- | --- | --- |
| **Whole sample** | | | |  |  |  |
| *APOE* ε4 carrier, *n* (%) | | | | 3 | 2 | 1 |
| Cognitive scores | | | |  |  |  |
|  | | | MoCA | 2 | 0 | 2 |
|  | | | RAVLT-total | 3 | 1 | 7 |
|  | | | RAVLT-delayed | 4 | 1 | 8 |
|  | | | WAIS-IV Coding | 7 | 4 | 8 |
| CSF markers | | | |  |  |  |
|  | | | Aβ42/40 ratio | 2 | 0 | 0 |
|  | | | p-tau181, pg/mL | 1 | 0 | 0 |
|  | | | NfL, pg/mL | 1 | 2 | 2 |
| **MRI subset** | | | |  |  |  |
| *APOE* ε4 carrier, *n* (%) | | | | 2 | 1 | 1 |
| Cognitive scores | | | |  |  |  |
|  | | MoCA | | 2 | 0 | 1 |
|  | | RAVLT-total | | 2 | 1 | 4 |
|  | | RAVLT-delayed | | 3 | 1 | 4 |
|  | | WAIS-IV Coding | | 3 | 4 | 4 |
| CSF markers | | | |  |  |  |
|  | Aβ42/Aβ40 ratio | | | 1 | 0 | 0 |
|  | p-tau181, pg/mL | | | 0 | 0 | 0 |
|  | NfL, pg/mL | | | 1 | 0 | 0 |

*Abbreviations*: *Aβ* amyloid-β, *Aβ42/40* amyloid-β 42/amyloid-β 40, *AD* Alzheimer’s disease, *CSF* cerebrospinal fluid, *MCI* mild cognitive impairment, *MoCA* Montreal Cognitive Assessment, *MRI* magnetic resonance imaging, *NfL* neurofilament light chain, *p-tau181* phosphorylated tau 181, *RAVLT* Rey Auditory Verbal Learning Test, *SCI* subjective cognitive impairment, *WAIS* Wechsler Adult Intelligence Scale

**Supplementary Table 2** Demographic, clinical, neuropsychological, CSF, and MRI data of non-demented (SCI and MCI) individuals by Aβ-positivity

|  | | **Non-demented Aβ-** | **Non-demented Aβ+** | **Test stat** | ***p*** | **Effect size** |
| --- | --- | --- | --- | --- | --- | --- |
| Age, years | | 59 (39–70) | 61 (46–72) | 3112.0*^U^* | 0.003 | 0.266 |
| Sex, female *n* (%) | | 84 (60.4) | 37 (60.7) | 8.908×10^-4χ2^ | 0.976 |  |
| Years of education | | 14 (9–23) | 13 (8.5–23) | 4985.0*^U^* | 0.047 | -0.176 |
| *APOE* ε4 carrier, *n* (%) | | 35 (25.9) | 50 (83.3) | 55.673^χ2^ | <0.001 |  |
| MCI, *n* (%) | | 24 (17.3) | 26 (42.6) | 14.538 ^χ2^ | <0.001 |  |
| Olfaction | |  |  |  |  |  |
|  | Total OID score | 15 (4–16) | 15 (5–16) | 4483.0*^U^* | 0.509 | -0.057 |
|  | Free OID score | 6 (0–14) | 4 (0–10) | 5318.5*^U^* | 0.004 | -0.255 |
|  | OD, *n* (%) | 12 (8.6) | 6 (9.8) | 0.075 | 0.784 |  |
| Cognitive scores | |  |  |  |  |  |
|  | MoCA | 26 (13–30) | 25 (18–30) | 4469.0*^U^* | 0.373 | -0.079 |
|  | RAVLT-total | 50 (12–71) | 46 (15–71) | 4696.0*^U^* | 0.072 | -0.162 |
|  | RAVLT-delayed | 11 (0–15) | 9 (0–15) | 4955.0*^U^* | 0.009 | -0.235 |
|  | WAIS-IV Coding | 54 (26–89) | 55 (14–93) | 3554.5*^U^* | 0.703 | 0.035 |
| CSF markers | |  |  |  |  |  |
|  | Aβ42/40 ratio | 1.05 (0.86–1.35) | 0.60 (0.32–0.85) | 8479.0*^U^* | <0.001 | -1.000 |
|  | p-tau181, pg/mL | 30 (14–79) | 61 (20–170) | 1145.0*^U^* | <0.001 | 0.728 |
|  | NfL, pg/mL | 640 (150–2280) | 830 (300–1850) | 2549.0*^U^* | <0.001 | 0.395 |
| MRI volumes, mL^*^ | |  |  |  |  |  |
|  | AMYG | 2.26 (0.18) | 2.16 (0.20) | 2.954^S^*^t^* | 0.004 | 0.539 |
|  | ENT | 4.65 (0.50) | 4.45 (0.53) | 2.087^S^*^t^* | 0.039 | 0.380 |
|  | HIP | 7.44 (0.59) | 7.21 (0.65) | 2.079^S^*^t^* | 0.040 | 0.379 |
|  | PHIP | 6.21 (0.60) | 6.01 (0.58) | 1.903^S^*^t^* | 0.059 | 0.347 |
|  | WM-hyper | 1.09 (0.00–52.94) | 1.88 (0.00–28.06) | 1538.0*^U^* | 0.029 | 0.231 |

Amyloid-positivity was defined as Aβ42/Aβ40[×10]<0.86. Mean (standard deviation) (continuous variables) and median (minimum–maximum) (discrete variables, and for continuous variables when parametric assumptions were not met) are shown. Effect sizes correspond to Cohen’s *d* for Student’s *t* and Welch’s *t* and rank-biserial correlation for Mann–Whitney *U*. ^S^*^t^*Student’s t-test was used, ^χ2^Pearson’s chi-squared test was used; *^U^*Mann–Whitney U test was used; ^*^MRI data was available for a subset of individuals (Aβ-, *n* = 105, Aβ+, *n* = 28).

Abbreviations: *Aβ* amyloid-β, *Aβ42/40* amyloid-β 42/amyloid-β 40, *AMYG* amygdala, *APOE* apolipoprotein E, *CSF* cerebrospinal fluid, *ENT* entorhinal cortex, *HIP* hippocampal, *MCI* mild cognitive impairment, *MoCA* Montreal Cognitive Assessment, *MRI* magnetic resonance imaging, *NfL* neurofilament light chain, *OD* olfactory dysfunction, *OID* odor identification, *PHIP* parahippocampal gyrus, *p-tau181* phosphorylated tau 181, *RAVLT* Rey Auditory Verbal Learning Test, *SCI* subjective cognitive impairment, *WAIS* Wechsler Adult Intelligence Scale, *WM-hyper* white matter hyperintensities

**Supplementary Table 3** Demographic, clinical, neuropsychological, CSF, and MRI data of all (SCI, MCI and AD) individuals by Aβ-positivity

|  | | **Non-demented Aβ-** | **All Aβ+** | **Test stat** | ***p*** | **Effect size** |
| --- | --- | --- | --- | --- | --- | --- |
| Age, years | | 59 (39–70) | 61 (46–72) | 5359.0*^U^* | 0.037 | 0.162 |
| Sex, female *n* (%) | | 84 (60.4) | 54 (58.7) | 0.069^χ2^ | 0.792 |  |
| Years of education | | 14 (9–23) | 13 (8.5–23) | 7334.0*^U^* | 0.058 | -0.147 |
| *APOE* ε4 carrier, *n* (%) | | 35 (25.9) | 70 (77.8) | 58.333^χ2^ | <0.001 |  |
| SCI, *n* (%) | | 115 (82.7) | 35 (38.0) |  |  |  |
| MCI, *n* (%) | | 24 (17.3) | 26 (28.3) |  |  |  |
| AD, *n* (%) | | NA | 31 (33.7) |  |  |  |
| Olfaction | |  |  |  |  |  |
|  | Total OID score | 15 (4–16) | 14 (4–16) | 7293.5*^U^* | 0.065 | -0.141 |
|  | Free OID score | 6 (0–14) | 4 (0–10) | 8245.5*^U^* | <0.001 | -0.289 |
|  | OD, *n* (%) | 12 (8.6) | 13 (14.1) | 1.733 | 0.188 |  |
| Cognitive scores | |  |  |  |  |  |
|  | MoCA | 26 (13–30) | 24 (9–30) | 7899.0*^U^* | <0.001 | -0.286 |
|  | RAVLT-total | 50 (12–71) | 37 (10–71) | 7590.5*^U^* | <0.001 | -0.335 |
|  | RAVLT-delayed | 11 (0–15) | 7.5 (0–15) | 7737.5*^U^* | <0.001 | -0.388 |
|  | WAIS-IV Coding | 54 (26–89) | 50 (4–93) | 5938.0*^U^* | 0.098 | 0.136 |
| CSF markers | |  |  |  |  |  |
|  | Aβ42/40 ratio | 1.05 (0.86–1.35) | 0.56 (0.30–0.85) | 12788.0*^U^* | <0.001 | -1.000 |
|  | p-tau181, pg/mL | 30 (14–79) | 68 (20–370) | 1178.0*^U^* | <0.001 | 0.814 |
|  | NfL, pg/mL | 640 (150–2280) | 900 (300–1910) | 3183.0*^U^* | <0.001 | 0.480 |
| MRI volumes, mL^*^ | |  |  |  |  |  |
|  | AMYG | 2.26 (0.18) | 2.12 (0.22) | 4.274^S^*^t^* | <0.001 | 0.707 |
|  | ENT | 4.65 (0.50) | 4.39 (0.55) | 2.947^S^*^t^* | 0.004 | 0.488 |
|  | HIP | 7.44 (0.59) | 7.04 (0.78) | 3.605^S^*^t^* | <0.001 | 0.596 |
|  | PHIP | 6.21 (0.60) | 5.93 (0.57) | 2.894^S^*^t^* | 0.004 | 0.479 |
|  | WM-hyper | 1.09 (0.00–52.94) | 1.96 (0.00–28.52) | 2147.0*^U^* | 0.024 | 0.217 |

Amyloid-positivity was defined as Aβ42/Aβ40[×10]<0.86. Mean (standard deviation) (continuous variables) and median (minimum–maximum) (discrete variables, and for continuous variables when parametric assumptions were not met) are shown. Effect sizes correspond to Cohen’s *d* for Student’s *t* and Welch’s *t* and rank-biserial correlation for Mann–Whitney *U*. ^S^*^t^*Student’s t-test was used, ^χ2^Pearson’s chi-squared test was used; *^U^*Mann–Whitney U test was used; ^*^MRI data was available for a subset of individuals (Aβ-, *n* = 105, Aβ+, *n* = 28).

Abbreviations: *Aβ* amyloid-β, *Aβ42/40* amyloid-β 42/amyloid-β 40, *AD* Alzheimer’s disease, *AMYG* amygdala, *APOE* apolipoprotein E, *CSF* cerebrospinal fluid, *ENT* entorhinal cortex, *HIP* hippocampal, *MCI* mild cognitive impairment, *MoCA* Montreal Cognitive Assessment, *MRI* magnetic resonance imaging, *NfL* neurofilament light chain, *OD* olfactory dysfunction, *OID* odor identification, *PHIP* parahippocampal gyrus, *p-tau181* phosphorylated tau 181, *RAVLT* Rey Auditory Verbal Learning Test, *SCI* subjective cognitive impairment, *WAIS* Wechsler Adult Intelligence Scale, *WM-hyper* white matter hyperintensities

|  | **All** | | **SCI** | | **MCI** | | **AD** | |
| --- | --- | --- | --- | --- | --- | --- | --- | --- |
|  | Total OID | Free OID | Total OID | Free OID | Total OID | Free OID | Total OID | Free OID |
| **Cognitive scores** |  |  |  |  |  |  |  |  |
| RAVLT-total | 0.075 | **0.241^*^** | -0.084 | 0.084 | 0.186 | 0.184 | 0.385 | **0.512^*^** |
| RAVLT-delayed | 0.105 | **0.233^*^** | -0.016 | 0.107 | 0.187 | 0.114 | 0.255 | **0.531^*^** |
| WAIS-IV Coding | **0.225^*^** | **0.163^*^** | 0.081 | -0.007 | 0.305**^†^** | 0.213 | 0.455**^†^** | 0.281 |
| **CSF markers** |  |  |  |  |  |  |  |  |
| Aβ42/40 ratio | 0.080 | **0.197^*^** | 0.043 | 0.108 | -0.208 | -0.092 | **0.419^*^** | 0.107 |
| p-tau181 | -0.044 | **-0.116^†^** | 0.011 | 0.032 | 0.139 | -0.068 | -0.170 | -0.107 |
| NfL | -0.087 | **-0.167^*^** | 0.044 | -0.033 | -0.075 | **-0.261^†^** | -0.202 | -0.330 |
| **MRI volumes** |  |  |  |  |  |  |  |  |
| AMYG | 0.022 | **0.160^†^** | -0.097 | 0.008 | 0.062 | 0.135 | 0.129 | 0.400 |
| ENT | 0.028 | 0.035 | -0.097 | -0.024 | 0.269 | 0.213 | -0.130 | -0.047 |
| HIP | **0.186^†^** | **0.212^**^** | 0.135 | 0.088 | 0.232 | 0.109 | **0.640^†^** | **0.720^**^** |
| PHIP | 0.013 | 0.003 | -0.034 | -0.028 | 0.010 | -0.033 | 0.242 | 0.054 |
| WM-hyper | -0.080 | -0.045 | 0.130 | 0.019 | **-0.541^**^** | -0.191 | -0.253 | -0.292 |

**Supplementary Table 4** Associations of cognitive performance CSF markers and MRI volumes with OID scores for the whole sample and stratified by diagnostic group additionally adjusting for *APOE* status

Spearman’s rho correlation coefficients are shown. **^†^*p*<0.05** (uncorrected significance threshold), **^*^*p*<0.0167**, **^**^*p*<0.010** (family-wise corrected significance thresholds).

*Abbreviations*: *Aβ* amyloid-β, *Aβ42/40* amyloid-β 42/amyloid-β 40, *AD* Alzheimer’s disease, *AMYG* amygdala, *CSF* cerebrospinal fluid, *ENT* entorhinal cortex, *HIP* hippocampal, *MCI* mild cognitive impairment, *MRI* magnetic resonance imaging, *NfL* neurofilament light chain, *OID* odor identification, *PHIP* parahippocampal gyrus, *p-tau181* phosphorylated tau 181, *SCI* subjective cognitive impairment, *WAIS* Wechsler Adult Intelligence Scale, *WM-hyper* white matter hyperintensities

**Supplementary Table 5** Associations of cognitive performance, CSF markers and MRI volumes with OID scores in non-demented Aβ-, non-demented Aβ+, and all Aβ+ groups additionally adjusting for *APOE* status

|  | **Non-demented Aβ-** | | **Non-demented Aβ+** | | **All Aβ+** | |
| --- | --- | --- | --- | --- | --- | --- |
|  | Total OID | Free OID | Total OID | Free OID | Total OID | Free OID |
| **Cognitive scores** |  |  |  |  |  |  |
| RAVLT-total | -0.039 | 0.041 | 0.077 | **0.426**^*^ | **0.270^*^** | **0.456^*^** |
| RAVLT-delayed | 0.019 | 0.111 | 0.114 | 0.254**^†^** | **0.275^*^** | **0.355^*^** |
| WAIS-IV Coding | 0.131**^†^** | 0.053 | 0.227 | 0.227 | **0.400^*^** | **0.323^*^** |
| **CSF markers** |  |  |  |  |  |  |
| Aβ42/40 | 0.093 | 0.086 | -0.010 | 0.138 | 0.122 | 0.150 |
| p-tau181 | 0.028 | 0.059 | 0.027 | -0.075 | -0.126 | -0.138 |
| NfL | -0.044 | -0.062 | -0.065 | -0.075 | **-0.182^†^** | **-0.189^†^** |
| **MRI volumes** |  |  |  |  |  |  |
| AMYG | -0.113 | -0.054 | **0.271^†^** | **0.332^†^** | 0.189 | **0.335**^**^ |
| ENT | 0.024 | 0.015 | 0.077 | 0.064 | 0.058 | 0.066 |
| HIP | 0.096 | 0.073 | 0.179 | 0.104 | 0.209 | **0.293^†^** |
| PHIP | 0.018 | -0.032 | -0.075 | -0.137 | -0.039 | -0.054 |
| WM-hyper | 0.110 | -0.009 | **-0.413**^**^ | -0.084 | **-0.367^**^** | -0.156 |

Non-demented Aβ- group includes Aβ- SCI and MCI individuals. Non-demented Aβ+ group includes Aβ+ SCI and MCI individuals. All Aβ+ group includes Aβ+ SCI, MCI and AD. Amyloid-positivity was defined as Aβ42/Aβ40[×10]<0.86. Spearman’s rho correlation coefficients are shown. **^†^*p*<0.05** (uncorrected significance threshold), **^*^*p*<0.0167**, **^**^*p*<0.010** (family-wise corrected significance thresholds).

*Abbreviations*: *Aβ* amyloid-β, *Aβ42/40* amyloid-β 42/amyloid-β 40, *AD* Alzheimer’s disease, *AMYG* amygdala, *APOE* apolipoprotein E, *CSF* cerebrospinal fluid, *ENT* entorhinal cortex, *HIP* hippocampal, *MCI* mild cognitive impairment, *MRI* magnetic resonance imaging, *NfL* neurofilament light chain, *PHIP* parahippocampal gyrus, *p-tau181* phosphorylated tau 181, *SCI* subjective cognitive impairment, *WAIS* Wechsler Adult Intelligence Scale, *WM-hyper* white matter hyperintensities

**Supplementary Table 6** Generalized additive models with OID performance as an outcome in the whole sample additionally adjusting for *APOE* status

|  | | Estimate | Std. Error | Test stat. | *p* |
| --- | --- | --- | --- | --- | --- |
| **Model: Total OID** | |  |  |  |  |
| Parametric terms | |  |  |  |  |
|  | (Intercept) | 19.116 | 2.417 | 7.908 | **7.31×10^-13^** |
|  | Age | -0.075 | 0.035 | -2.150 | 0.033 |
|  | Sex | -1.079 | 0.393 | -2.743 | **0.007** |
|  | Years of education | 0.055 | 0.066 | 0.836 | 0.404 |
|  | *APOE* status | -0.013 | 0.441 | -0.031 | 0.976 |
| Smooth terms | |  |  |  |  |
|  | Aβ42/40 ratio | - | - | 0.830 | 0.364 |
|  | HIP volume | - | - | 3.830 | 0.052 |
| **Model: Free OID** | |  |  |  |  |
| Parametric terms | |  |  |  |  |
|  | (Intercept) | 12.718 | 2.736 | 4.649 | **5.73×10^-6^** |
|  | Age | -0.097 | 0.040 | -2.463 | **0.015** |
|  | Sex | -2.208 | 0.448 | -4.925 | **2.39×10^-6^** |
|  | Years of education | 0.102 | 0.074 | 1.366 | 0.174 |
|  | *APOE* status | -0.345 | 0.502 | -0.687 | 0.493 |
| Smooth terms | |  |  |  |  |
|  | Aβ42/40 ratio | - | - | 2.977 | 0.087 |
|  | p-tau181 | - | - | 0.107 | 0.744 |
|  | HIP volume | - | - | 5.302 | **0.023** |

Parametric terms are reported with *t*-statistics, and smooth terms with *F*-statistics.

*Abbreviations*: *Aβ* amyloid-β, *Aβ42/40* amyloid-β 42/amyloid-β 40, *AD* Alzheimer’s disease, *HIP* hippocampal, *OID* olfactory identification, *p-tau181* phosphorylated tau 181
